# Trends in unawareness of cardiovascular disease risk factors among racial & ethnic groups in the United States

**DOI:** 10.1016/j.ajpc.2026.101456

**Published:** 2026-01-30

**Authors:** Ramzi Ibrahim, Kamal Awad, Abdelrahman Hafez, Hoang Nhat Pham, Min Choon Tan, Mohammed Salih, Justin Z Lee, Dan Sorajja, Luis R Scott, Chadi Ayoub, Reza Arsanjani

**Affiliations:** a Department of Cardiovascular Medicine, Mayo Clinic, Phoenix, AZ, USA; b Department of Cardiovascular Medicine, Mayo Clinic, Rochester, MN, USA; c Department of Medicine, University of Arizona, Tucson, AZ, USA; d Department of Cardiology, Baylor Scott and White-The Heart Hospital, Plano, TX, USA; e Department of Cardiovascular Medicine, Cleveland Clinic, Cleveland, OH, USA

Cardiovascular disease remains a leading cause of morbidity and mortality in the United States [1]. Timely identification and management of risk factors such as hypertension, hyperlipidemia, and diabetes are critical to improving outcomes [1]. Recent data suggest rising hypertension unawareness, particularly among younger adults and women [2]. However, racial disparities in awareness of these risk factors remain poorly characterized. We aimed to examine temporal trends in awareness among non-Hispanic Black, non-Hispanic White, and Hispanic adults using nationally representative data.

We analyzed data from the National Health and Nutrition Examination Survey (NHANES), a nationally representative survey that combines interviews, physical examinations, and laboratory testing to assess health status in the U.S. population. This study did not require ethical board approval given the use of publicly available, anonymous data, in accordance with the Common Rule.

Adults aged ≥18 years with at least one cardiovascular risk factor, hypertension, hyperlipidemia, or diabetes, were included. Diabetes was defined as fasting glucose ≥126 mg/dL or hemoglobin A1c ≥6.5 %; hypertension as systolic BP ≥140 mmHg or diastolic BP ≥90 mmHg (averaged over three readings), or antihypertensive use; and hyperlipidemia as total cholesterol ≥240 mg/dL [3]. Participants were considered “unaware” if they met objective criteria but reported never being told they had the condition by a healthcare professional on survey questionnaires. We applied a uniform definition of hypertension across all NHANES cycles. Although guideline thresholds were lowered in 2017, we intentionally did not adopt the newer criteria in later cycles, as doing so would have introduced artificial shifts in prevalence and awareness unrelated to true changes in detection or clinical recognition. This approach is consistent with prior NHANES trend analyses and allows observed changes in unawareness to more accurately reflect secular trends rather than definitional artifacts.

Temporal trends in percentages of unawareness from 2013 to 2023 were assessed using survey-weighted linear regression models. This was repeatedly done for non-Hispanic Black, non-Hispanic White, and Hispanic adults within each of the three cardiovascular disease risk factors. All analyses applied NHANES sampling weights to ensure national representativeness. R software (v 4.3.2, R Foundation for Statistical Computing) was used for statistical analysis.

A total of 25,467 adults with at least one cardiovascular risk factor were included; 53 % were female, and the median age was 51 years (IQR 34–66). The cohort comprised 6119 non-Hispanic Black adults, 12,559 non-Hispanic White adults, and 6789 Hispanic adults.

Among non-Hispanic Black adults, hypertension unawareness declined slightly from 16.09 % (95 % CI, 13.68–18.51) in 2013–2014 to 13.71 % (95 % CI, 10.86–16.57) in 2021–2023, with no significant linear temporal trend observed (p-trend=0.7; Fig. 1A). Diabetes unawareness remained largely stable, from 27.78 % (95 % CI, 18.89–36.66) in 2013–2014 to 25.44 % (95 % CI, 18.39–32.49) in 2021–2023 (p-trend=0.3; Fig. 1B). Hyperlipidemia unawareness increased from 45.45 % (95 % CI, 36.44–54.45) in 2013–2014 to 54.10 % (95 % CI, 32.24–75.96) in 2021–2023, though this trend was not statistically significant (p-trend=0.4; Fig. 1C).

**Fig. 1:**
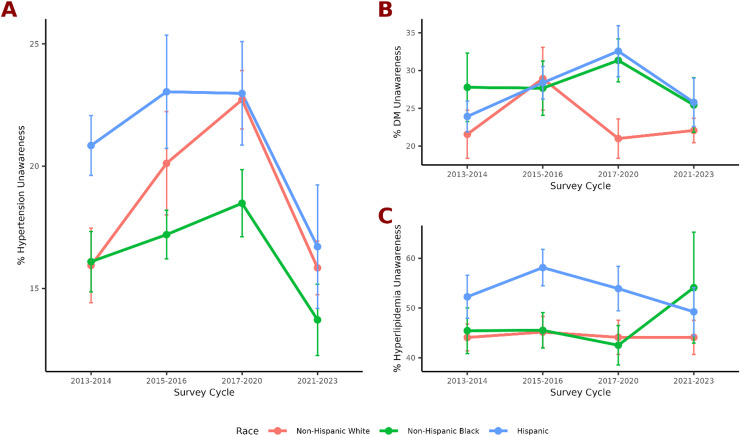
Line plots showing changes in percentages of unawareness according to race for (A) hypertension, (B) diabetes, and (C) high cholesterol across the survey cycles.

Among non-Hispanic White adults, hypertension unawareness remained stable from 15.94 % (95 % CI, 12.96–18.92) in 2013–2014 to 15.84 % (95 % CI, 13.70–17.98) in 2021–2023, with no evidence of a significant temporal trend (p-trend=0.4). Diabetes unawareness also remained stable, from 21.57 % (95 % CI, 15.33–27.81) in 2013–2014 to 22.08 % (95 % CI, 18.94–25.21) in 2021–2023 (p-trend>0.9). Hyperlipidemia unawareness was unchanged, from 44.08 % (95 % CI, 38.81–49.34) in 2013–2014 to 44.10 % (95 % CI, 37.38–50.82) in 2021–2023 (p-trend=0.7).

Among Hispanic adults, hypertension unawareness declined from 20.84 % (95 % CI, 18.44–23.24) in 2013–2014 to 16.71 % (95 % CI, 11.76–21.65) in 2021–2023, although the overall temporal trend was not statistically significant (p-trend=0.14). Diabetes unawareness increased modestly from 23.93 % (95 % CI, 19.93–27.92) in 2013–2014 to 25.79 % (95 % CI, 19.55–32.03) in 2021–2023 (p-trend=0.6). Hyperlipidemia unawareness decreased from 52.26 % (95 % CI, 43.78–60.74) in 2013–2014 to 49.24 % (95 % CI, 39.74–58.75) in 2021–2023, with no significant linear trend over time (p-trend=0.5).

In this nationally representative analysis, we found that hypertension unawareness remained largely stable from 2013 to 2023, with heterogeneity across racial and ethnic groups. Descriptively, hypertension unawareness remained stable among non-Hispanic White adults and declined modestly among non-Hispanic Black and Hispanic adults; however, no statistically significant overall temporal trends were observed based on survey-weighted regression analyses. Similarly, unawareness of diabetes and hyperlipidemia was generally stable over time across racial and ethnic groups, although persistently high levels of unawareness were observed across populations.

Our findings should be interpreted in the context of prior NHANES-based analyses, particularly the study by Fan et al., which reported substantial improvements in hypertension awareness from 1999 through 2013–2014, followed by a decline by 2017–2018 [4]. Fan et al. identified a post-2013 inflection point, with awareness peaking in 2013–2014 and subsequently decreasing across multiple demographic subgroups, including non-Hispanic White and non-Hispanic Black adults. While our results do not demonstrate a consistent worsening of hypertension unawareness in the most recent decade, they extend the NHANES evidence base into 2021–2023 and suggest that earlier gains in hypertension detection may have plateaued rather than continued to improve. Differences in outcome framing (awareness vs unawareness), analytic windows, and modeling approaches likely contribute to variation across studies, and indicate that progress in hypertension recognition may be uneven and vulnerable to disruption across population subgroups.

Hypertension affects nearly half of U.S. adults [5], and persistent unawareness remains a critical public health concern given the importance of early diagnosis and treatment [1]. Although temporal changes in diabetes and hyperlipidemia unawareness were not pronounced in our analysis, rates remained substantial, often exceeding 20 %, highlighting ongoing gaps in cardiovascular risk factor recognition. The observed differences in unawareness patterns across conditions likely reflect distinct screening pathways. Hypertension is frequently asymptomatic and often identified opportunistically during routine clinical encounters, whereas diabetes screening has become increasingly standardized and laboratory-driven, supported by hemoglobin A1c testing and integration into preventive care and chronic disease management programs. In contrast, recognition of hyperlipidemia depends heavily on access to routine laboratory testing and longitudinal follow-up, which may contribute to persistently high levels of unawareness in some populations.

Limitations of this study include declining NHANES response rates over time and potential recall bias from self-reported awareness. Additionally, limited sample sizes precluded stable trend analyses in some racial and ethnic groups beyond those included in this study. Nonetheless, these nationally representative data highlight persistent gaps in cardiovascular risk factor recognition and highlight the need for sustained, population-level strategies to improve screening, awareness, and engagement in preventive care across diverse populations.

## Author agreement form

We wish to confirm that there are no known conflicts of interest associated with this publication and there has been no significant financial support for this work that could have influenced its outcome.

We confirm that the manuscript has been read and approved by all named authors and that there are no other persons who satisfied the criteria for authorship but are not listed. We further confirm that the order of authors listed in the manuscript has been approved by all of us.

We confirm that we have given due consideration to the protection of intellectual property associated with this work and that there are no impediments to publication, including the timing of publication, with respect to intellectual property. In so doing we confirm that we have followed the regulations of our institutions concerning intellectual property.

We understand that the Corresponding Author is the sole contact for the Editorial process (including Editorial Manager and direct communications with the office). He is responsible for communicating with the other authors about progress, submissions of revisions and final approval of proofs. We confirm that we have provided a current, correct email address which is accessible by the Corresponding Author.

Signature of Corresponding Author on Behalf of All Authors:

Ramzi Ibrahim, 10/25/2025.

## Funding statement

This research did not receive any specific grant from funding agencies in the public, commercial, or not-for-profit sectors.

## Conflict of interest

Authors have no conflict of interest.

## CRediT authorship contribution statement

**Ramzi Ibrahim:** Writing – review & editing, Writing – original draft, Visualization, Validation, Supervision, Resources, Project administration, Methodology, Formal analysis, Data curation, Conceptualization. **Kamal Awad:** Writing – original draft, Visualization, Supervision, Software, Resources, Project administration, Methodology, Formal analysis, Data curation, Conceptualization. **Abdelrahman Hafez:** Writing – original draft, Supervision, Software, Project administration, Methodology, Formal analysis, Data curation, Conceptualization. **Hoang Nhat Pham:** Writing – original draft, Visualization, Supervision, Software, Project administration, Methodology, Formal analysis, Data curation, Conceptualization. **Min Choon Tan:** Writing – original draft, Visualization, Validation, Supervision, Software, Project administration, Methodology, Data curation, Conceptualization. **Mohammed Salih:** Writing – review & editing, Writing – original draft, Validation, Supervision, Resources, Project administration, Methodology, Formal analysis, Data curation, Conceptualization. **Justin Z Lee:** Writing – review & editing, Writing – original draft, Visualization, Validation, Software, Resources, Project administration, Investigation, Data curation, Conceptualization. **Dan Sorajja:** Writing – original draft, Visualization, Supervision, Software, Resources, Project administration, Investigation, Formal analysis, Data curation, Conceptualization. **Luis R Scott:** Writing – review & editing, Writing – original draft, Visualization, Supervision, Software, Methodology, Investigation, Data curation, Conceptualization. **Chadi Ayoub:** Writing – original draft, Visualization, Validation, Software, Project administration, Methodology, Formal analysis, Data curation, Conceptualization. **Reza Arsanjani:** Writing – review & editing, Visualization, Supervision, Software, Project administration, Investigation, Formal analysis, Data curation, Conceptualization.

## Declaration of competing interest

The authors declare that they have no known competing financial interests or personal relationships that could have appeared to influence the work reported in this paper.
